# High leptin levels in blood are associated with chronic widespread pain in rheumatoid arthritis

**DOI:** 10.1186/s13075-024-03463-x

**Published:** 2024-12-23

**Authors:** M. Aronsson, S. Bergman, E. Lindqvist, M. L. E. Andersson

**Affiliations:** 1 Department of Clinical Sciences Lund, Section of Rheumatology, Lund University, Faculty of Medicine, Lund, Sweden; 2 Spenshult Research and Development Center, Bäckagårdsvägen 47, Halmstad, 302 74 Sweden; 3 Department of Rheumatology, Capio Movement, Halmstad, Sweden; 4 School of Public Health and Community Medicine / Primary Health Care, Institute of Medicine, Sahlgrenska Academy, University of Gothenburg, Gothenburg, Sweden; 5 Skåne University Hospital, Department of Rheumatology, Lund, Sweden

**Keywords:** CWP, Adipokines, Biomarkers, Sensitization, RA

## Abstract

**Background:**

High body mass index (BMI) has been shown to have an association with chronic widespread pain (CWP), both in Rheumatoid arthritis (RA) and in other pain conditions such as fibromyalgia. Research on the adipose tissue and it’s adipokines, for example the well described leptin, is emerging. The objective of this study was to determine if there is an association between leptin levels in blood and CWP in patients with RA.

**Methods:**

In this cross-sectional study 334 patients with RA filled in a questionnaire including a pain mannequin and questions on weight, length and waist circumference. Data from the pain mannequin was used to calculate CWP. The subjects also submitted blood samples to a biobank that were used for this study to determine leptin levels using an ELISA-method.

**Results:**

Patients fulfilling the 2019 criteria for CWP in this study had significantly higher leptin levels, waist circumferences and BMI. There was a significant association between leptin levels and CWP, odds ratio (OR) 1.014 (95% confidence interval (CI) 1.007–1.020), *p* < 0.001. The association remained after adjusting for BMI, gender and age OR 1.008 (95% CI 1.000-1.017), p 0.046. When leptin was divided into quartile groups a trend could be observed where higher leptin values gave higher OR for CWP.

**Conclusions:**

This study showed an increased occurrence of CWP in RA-patients with high leptin levels. The association between leptin and CWP remained after adjusting for gender, age and BMI or waist circumference. This suggests that not just production of leptin in the adipose tissue, but also other factors such as leptin resistance may play a role. The association between leptin and CWP was strongest in the patients with the highest leptin levels.

**Trial registration:**

This study is a cross-sectional study without intervention and the cohort used was initiated prior to the implementation of mandatory registration requirements, therefore it is not registered.

## Background

In the last 20 years there has been a revolution in the pharmacological treatment of inflammation in reumatoid arthritis (RA). Despite this, chronic pain remains a substantial difficulty in RA, supposedly caused by peripheral and central sensitization [1]. Around 20% of RA patients suffers from concomitant fibromyalgia and 30% of patients fulfils the 1990 criteria for chronic widespread pain (CWP) [2–5]. RA patients with high scores of fibromyalgianess exhibits similar patterns on functional MRI as fibromyalgia-patients without RA, suggesting similar patterns of central sensitization [6]. To better classify the patients with severe widespread pain a new, more stringent, definition of CWP was recently developed [7]. In the new definition, WP2019, a patient must have pain in at least 4 of 5 body regions (left upper, left lower, right upper, right lower, axial) and have at least 7 painful sites out of 15 to be classified as having CWP [7].

Multiple studies have confirmed that RA-patients with high body mass index (BMI) and metabolic syndrome have more disease activity and pain [8–11]. How the adipose tissue interacts with the immune system in autoimmune diseases such as RA is under investigation and in recent years the research on the adipose tissue has expanded from research on cardiovascular disease and diabetes to encompass its role as an endocrine organ with an important function in metabolism and inflammation at large [12].

Leptin is a peptide hormone produced predominantly in the adipose tissue, but also expressed in a variety of other tissues including bone marrow, lymphoid tissues, skeletal muscle and cartilage [13, 14]. As described today, the best-known leptin receptor signal transduction goes through the JAK/STAT system via cytoplasmatic JAK2 [14]. Leptin regulate energy homeostasis but is also involved in neuroendocrine function, insulin resistance and is considered pro-inflammatory [12, 13]. Leptin concentration is affected by the amount of body fat weight and sex, where women have higher concentrations, likely because of difference in sex hormones [13]. Reports have suggested higher leptin levels in patients with fibromyalgia compared to age, sex and BMI matched controls and that fibromyalgia patients tend to demonstrate an overexpression of leptin independent of the contribution of their overweight [15, 16]. A small study showed that pain intensity from day-to-day varied with leptin concentration in women with fibromyalgia [17]. The question if leptin has a connection to the process of central sensitization has been investigated mostly in vitro and in animal models. For example, there are theories that activation of microglia in the dorsal root and spinal cord is associated with pain sensitization and leptin has been shown to affect the cytokine release from microglia in vitro [18, 19]. Leptin receptors have also been found intracellularly in the dorsal root ganglia of rats where their expression could be upregulated by administration of oestrogen [20]. Another study showed that spinal administration of leptin antagonists prevented and reversed neuropathic pain behaviours in rats in a sciatic nerve injury model [21].

The aim of this study was to investigate if there is an association between leptin levels and chronic widespread pain in patients with RA.

## Method

### Patients

This cross-sectional study based on a questionnaire and lab data consists of 334 RA-patients from the longitudinal multicentre cohort Better Anti-Rheumatic PharmacoTherapy (BARFOT) [22] The BARFOT cohort included patients with early RA (< 1 year duration) between 1992 and 2006 and followed them for 15 years. In 2010 a questionnaire was sent out to 1910 eligible patients, 1534 responded which gave an 80% response rate. Out of these 1534 patients, 334 submitted blood samples according to the study protocol for the BARFOT-study within one year from the completion of the questionnaire. These 334 patients were selected for the current study. The patients selected for the study were included in the BARFOT cohort between 1994 and 2006, the median being 2002. Their mean disease duration at the point of the questionnaire was 8 (Standard deviation (SD) 4,1) years.

### Questionnaire

The questionnaire included inquiries on weight, height, waist circumference (with clear instructions on how to measure), pharmacological treatment, smoking, alcohol (AUDIT-C) [23] and a pain mannequin. The mannequin showed 18 predefined regions [24]. The responses of the pain mannequin were used to calculate CWP with an adapted version of WP2019, here called CWP2019, where the knees and anterior chest of our pain mannequin were excluded from analysis to match the 15 sites in WP2019 [7]. CWP2019 was determined as pain ≥ 3 months in at least 4 of 5 main body regions (the spine, the upper and the lower quadrants of the body), with at least 7 painful sites out of 15 possible.

### Registry data

Inclusion date in the BARFOT cohort, date of the questionnaire, rheumatoid factor (RF) and anti-citrullinated protein antibodies (ACPA) status were retrieved from the BARFOT study database.

### Laboratory work

Blood samples from the BARFOT cohort biobank were analysed using an enzyme-linked immunosorbent assay ELISA, (Alpco) in accordance with the manufacturer’s instructions.

### Statistics

**Fig. 1:**
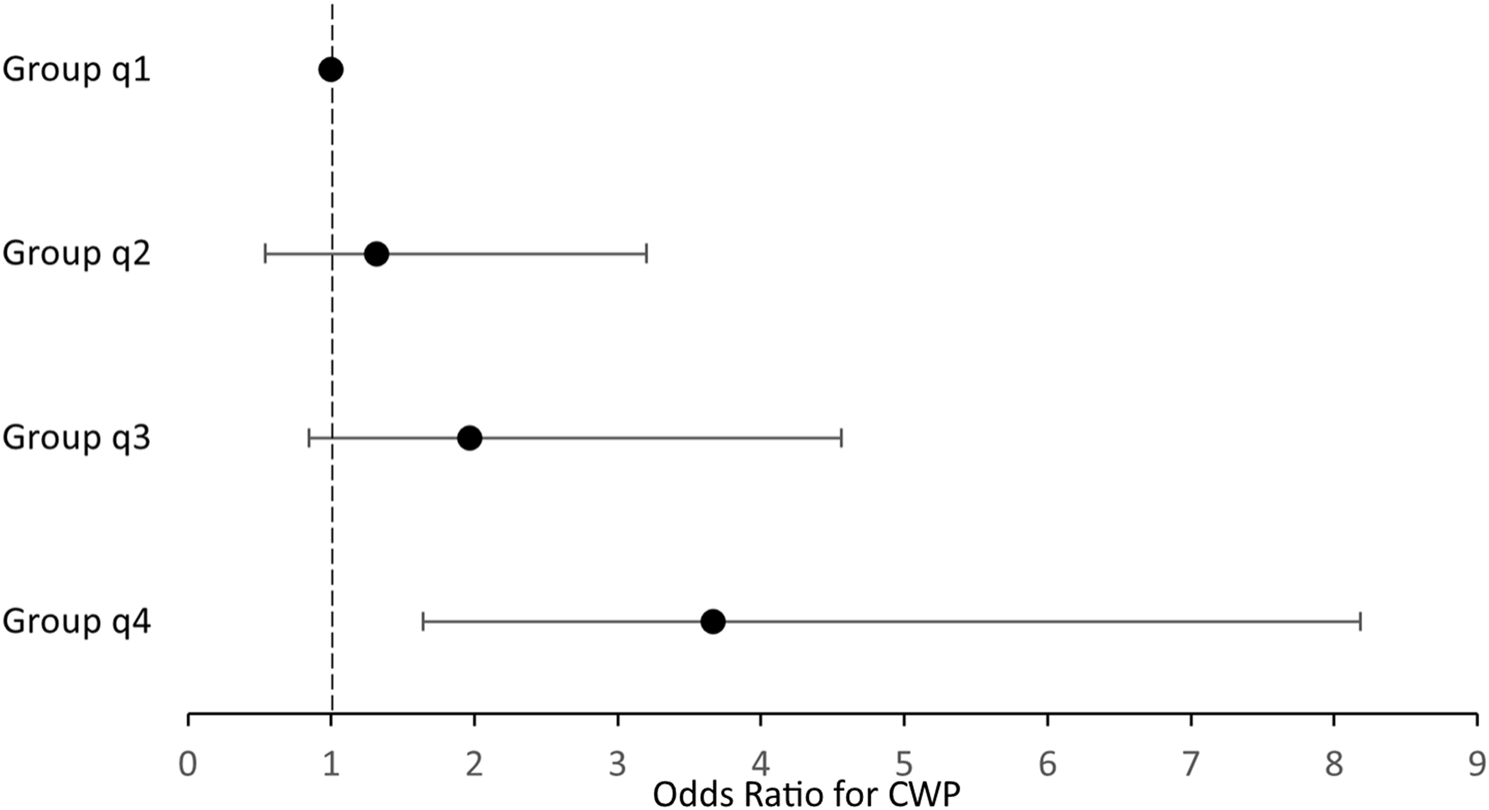
Associations between leptin levels and CWP2019 by logistic regression model. Odds ratio with confidence intervals. The leptin levels are divided into quartiles were the lowest quartile (Group q1) is the reference

Statistical analyses have been made using SPSS statistics 29 software, (IBM). The level of significance in the analyses was set to < 0.05. Since many of the variables were not normally distributed, mostly non-parametric methods were used. The calculations were made with complete case analyses without imputations. Comparisons between patients with and without CWP2019 were made with Mann-Whitney U test and Pearson’s chi-square test. Ficher’s exact test was used if *n* < 5. To describe the participants disease duration at the time of the questionnaire mean and standard deviation were used, for other continuous variables median and the first and third quartile were used as dispersion measures. To investigate associations between leptin and CWP2019 simple and multiple logistic regression were used. For one of the simple logistic regressions the leptin values were divided into 4 groups based on their quartiles, Fig. 1. To estimate the sufficient number of participants in the study we hypothesised that the association would resemble the one previously reported in patients with knee pain, where a significant association between leptin and CWP2019 could be found in less than 300 participants [25]. A power calculation revealed that around 70 patients with CWP2019 and 70 without CWP2019 would be sufficient to demonstrate a significant difference between groups with an effect size of 0.5 and 90% power.

## Results

In this study 69 of 334 patients (21%) fulfilled the 2019 criteria for chronic widespread pain. The patients with CWP2019 had significantly higher leptin levels, BMI and waist circumference compared with the patients without CWP2019, Table 1. The patients with CWP2019 reported more VAS pain, VAS fatigue, tender and swollen joints and had worse HAQ-scores, Table 1. RF and ACPA negativity as well as female gender were more common in the group with CWP2019 but there was no difference in age between groups, Table 1.

**Table 1:** Characteristics for patients with and without chronic widespread pain, CWP2019. Presented values are median (q1-q3) or numbers (%)

	Missing* CWP/ No CWP	All *N* = 334	CWP *n* = 69	No CWP *n* = 264	*p* value
Age, years	0/0	64 (55–73)	64 (56–74)	65 (55–73)	0,841
Gender, female	0/0	236 (71)	57 (83)	178 (67)	0.014
RF positive	0/0	205 (61)	34 (49)	171 (65)	0.018
ACPA positive	5/27	188 (62)	29 (45)	159 (67)	0.001
Swollen joints	2/7	1 (0–5)	5 (2–10)	1 (0–3)	< 0.001
Tender joints	1/6	3 (1–9)	12 (5–18)	2 (0–5)	< 0.001
HAQ (0–3)	0/0	0.38 (0-0.88)	1.13 (0.56–1.50)	0.25 (0-0.75)	< 0.001
VAS pain (0-100)	0/12	30 (20–60)	60 (40–70)	30 (10–50)	< 0.001
VAS fatigue (0-100)	0/5	40 (20–70)	70 (50–80)	30 (10–60)	< 0.001
BMI	3/9	25.40 (22.64–28.15)	27.23 (23.73–30.81)	24.75 (22.60-27.68)	0.002
Waist circumference, cm	20/57	91 (83–100)	96 (85–106)	90 (83–99)	0.051
Leptin ng/ml	0/0	17.48 (7.0-39.59)	29.84 (10.84–60.84)	13.91 (6.41–33.47)	< 0.001

CWP, widespread pain according to 2019 criteria; RF, rheumatoid factor; ACPA; anti-citrullinated protein antibodies, HAQ, Health Assessment Questionnaire; VAS, visual analogue scale; BMI, body mass index; Swollen joints: self-reported 28 swollen joint count; Tender joints: self-reported 28 tender joint count; p value, calculated between groups CWP and No CWP; *one patient did not fill in the pain mannequin so no CWP2019 could be calculated

There was an association between leptin levels and CWP2019, odds ratio (OR) 1.014 (95% conference interval (CI) 1.007–1.020), *p* < 0.001. The association remained significant after adjusting for BMI, gender and age OR 1.008 (95% CI 1.000-1.017), p 0.046, Table 2. When adjusting for gender, age and waist circumference instead of BMI there was also a significant association between leptin levels and CWP2019, Table 2. When leptin was divided into quartile groups, a simple logistic regression revealed a trend where higher leptin values gave higher OR for CWP2019, Fig. 1.

**Table 2:** The association between leptin and having CWP2019, adjusted for age, gender, BMI and waist circumference. Simple logistic regression and multiple logistic regression, OR (95% CI)

	Simple and multiple logistic regression for having CWP2019		
	Model 1	Model 2	Model 3
Leptin (ng/ml)	1.014 (1.007–1.120)	1.008 (1.000-1.017)	1.010 (1.001–1.019)
Age		1.003 (0.982–1.024)	0.989 (0.964–1.014)
Gender, female		1.805 (0.861–3.782)	2.366 (0.898–6.234)
BMI		1.053 (0.979–1.132)	
Waist circumference (cm)			1.021 (0.992–1.051)
No	333	321	256

CWP2019: Chronic Widespread Pain according to 2019 definition; BMI: body mass index; Age: age at time of questionnaire. No, number included in model

Smoking or alcohol use did not affect the association between leptin and CWP2019, Table 3. Self-reported swollen joint count had an association with CWP2019 but could not explain the association between leptin and CWP2019, Table 3.

**Table 3:** The effect of smoking, alcohol and swollen joints respectively on the association between leptin and having CWP2019. Multiple logistic regressions, OR (95% CI)

Multiple logistic regressions for having CWP2019			
	Model 1	Model 2	Model 3
Leptin (ng/ml)	1.011(1.002–1.020)	1.010 (1.002–1.018)	1.012 (1.004–1.019)
Smoking	0.586 (0.261–1.316)		
High AUDIT C, 0–12		0.538 (0.234–1.240)	
Swollen joints			1.147 (1.090–1.207)
No	178	257	324

CWP2019: Chronic Widespread Pain according to 2019 definition; High AUDIT C: AUDIT C ≥ 4 for women and ≥ 5 for men indicating risk consumption; Swollen joints: self-reported 28 swollen joint count; No, number included in model

Regarding pharmacological treatment, patients with CWP2019 used less methotrexate and more corticosteroids and NSAIDs than the patients without CWP2019. Regarding earlier treatment experience with methotrexate, there was no significant difference between the groups, Table 4. The association between leptin and CWP2019 remained when adjusting for different pharmacological treatments, Table 5.

**Table 4:** Pharmacological treatment. Drugs with > 10 users included in table. Presented values are numbers (%)

	Missing* CWP/No CWP	CWP *n* = 69	No CWP *N* = 264	*p* value
Methotrexate	1/4	34 (50)	167 (64)	0.032
Sulfasalazine	1/5	10 (15)	33 (13)	0.670
Antimalarial drugs	1/4	4 (6)	15 (6)	0.972
TNF inhibitors	1/4	12 (18)	51 (20)	0.714
Corticosteroids	1/3	33 (49)	62 (24)	< 0.001
NSAIDs	0/2	27 (39)	70 (27)	0.044
Earlier treatment Methotrexate	5/16	44 (69)	165 (67)	0.737

CWP, widespread pain according to 2019 criteria; TNF inhibitors, tumour necrosis factor inhibitor; NSAID, nonsteroidal anti-inflammatory drugs. *One patient did not fill in the pain mannequin so no CWP2019 could be calculated

**Table 5:** The effect of pharmacological treatments respectively on the association between leptin and having CWP2019. Multiple logistic regressions with OR (95% CI)

Multiple logistic regressions for having CWP2019						
	Model 1	Model 2	Model 3	Model 4	Model 5	Model 6
Leptin (ng/ml)	1.014 (1.007–1.021)	1.014 (1.007–1.020)	1.014 (1.007–1.021)	1.014 (1.007–1.020)	1.013 (1.006–1.020)	1.014 (1.007–1.021)
Methotrexate	0.521 (0.297–0.912)					
Sulfasalazine		1.186 (0.532–2.643)				
Antimalarial			1.184 (0.376–3.730)			
TNF inhibitors				0.888 (0.430–1.836)		
Corticosteroids					2.829 (1.596–5.014)	
NSAIDs						1.939 (1.087-3,462)
No	328	327	328	328	329	331

CWP2019: Chronic Widespread Pain according to 2019 definition; TNF inhibitors, tumour necrosis factor inhibitor; NSAID, nonsteroidal anti-inflammatory drugs, No, number included in model

## Discussion

In this study we found an association between leptin levels and chronic widespread pain in RA. The association was strongest for the patients with the highest leptin levels and remained significant after adjusting for gender, age and BMI or waist circumference. The fact that the association remained when adjusting for waist circumference, raises the thought that there could be processes other than leptin production in adipocytes that maintain high leptin levels in these individuals. The concept of leptin resistance has been studied mostly in the field of obesity but is also thought to affect cognitive abilities and depression [26]. Leptin resistance is thought to derive from three different mechanisms: saturated leptin transport over the blood brain barrier, downregulation of leptin receptors and problems in the downstream signalling cascade through the JAK/STAT via the JAK 2 pathway [26].

How the concept of leptin resistance affects the experience of pain and central sensitization remains to be explored. Interestingly, our research group found the same pattern of association between leptin levels and CWP2019 in a cohort of individuals with knee pain (without rheumatologic disease), implying an association more connected to pain than to inflammation [25]. This is supported by the fact that the association between leptin levels and CWP2019 in the current study remained after adjusting for swollen joints, which could be viewed as a substitution marker for inflammation. In a study from 2007 where leptin levels were analysed in 30 patients with RA and 30 patients in a control group with osteoarthritis, the authors could not find any difference in leptin levels between groups [27], possibly because both these groups commonly have chronic pain and raised leptin levels? The association between leptin and CWP2019 remained after adjusting for various pharmacological treatments where some of them, for example TNF inhibitors, have potent anti-inflammatory properties. This supports the notion that concepts other than classic inflammation could be involved.

A weakness in the current study is the use of existing blood samples from the BARFOT biobank. The samples were not collected the same day as the questionnaire, and we have no records of what time of day the samples were drawn. In our knee pain study cited above however, where we found very similar results on the association between leptin and CWP2019 all the leptin samples were drawn fasting in the morning. Since we have a relatively large number of participants in the current study the effect of day-to-day variation in leptin levels is probably minor.

To our knowledge no other studies have examined the relationship between leptin and chronic widespread pain in RA and a strength of the current study is that a relatively large number of RA patients could be studied. In the emerging field of adipokine-studies, performed mostly in vitro and animal models, this study could act hypothesis generating in the continuous investigation of leptin’s effects in the central nervous system, dorsal root ganglia and peripheral tissue. The connection between leptin and the JAK-STAT system is especially interesting since studies have shown better effect on pain in RA-patients with JAK inhibitors such as Baricitinib (JAK 1 and 2 inhibitor) than with adalimumab, possibly through changes in activation of microglia in the spinal cord and suppression of the JAK/STAT system in the dorsal root ganglia [28–30]. In this study the adipokine leptin was investigated, but the intricate interplay between different adipokines such as adiponectin resistin and visfatin warrants additional studies and the other adipokines eventual associations to CWP and sensitization needs to be further explored.

In conclusion, the findings in this study encourage further investigation of the leptin system and its possible connection to central sensitization and pain. The current study supports an association between leptin levels and chronic widespread pain even after adjusting for BMI or waist circumference. The results need to be confirmed in other studies but raises hope for future possible treatment options in the adipokine system for people with RA and chronic widespread pain unresponsive to modern immunosuppressive medication.

## Data Availability

No datasets were generated or analysed during the current study.

## Abbreviations

ACPA: Anti-Citrullinated Protein Antibodies
AUDIT-C: Alcohol Use Disorders Identification Test-Concise
BARFOT: Better Anti-Rheumatic PharmacoTherapy
BMI: Body Mass Index
CI: Confidence Interval
CWP: Chronic Widespread Pain
CWP2019: Modified Chronic Widespread Pain According to 2019 criteria
ELISA: Enzyme-Linked Immunosorbent Assay
HAQ: Health Assessment Questionnaire
JAK/STAT: Janus Kinase/Signal Transducers and Activators of Transcription
OR: Odds Ratio
RA: Rheumatoid Arthritis
RF: Rheumatoid Factor
SD: Standard Deviation
VAS: Visual Analogue Scale
WP2019: Chronic Widespread Pain According to 2019 criteria
